# An FPGA-Based WASN for Remote Real-Time Monitoring of Endangered Species: A Case Study on the Birdsong Recognition of *Botaurus stellaris*

**DOI:** 10.3390/s17061331

**Published:** 2017-06-08

**Authors:** Marcos Hervás, Rosa Ma Alsina-Pagès, Francesc Alías, Martí Salvador

**Affiliations:** GTM—Grup de recerca en Tecnologies Mèdia, La Salle-Universitat Ramon Llull, C/Quatre Camins 30, 08022 Barcelona, Spain; ralsina@salleurl.edu (R.M.A.-P.); falias@salleurl.edu (F.A.); martisalvad@gmail.com (M.S.)

**Keywords:** wireless acoustic sensor networks, FPGA, remote real-time monitoring, birdsong recognition, endangered species, *Botaurus stellaris*

## Abstract

Fast environmental variations due to climate change can cause mass decline or even extinctions of species, having a dramatic impact on the future of biodiversity. During the last decade, different approaches have been proposed to track and monitor endangered species, generally based on costly semi-automatic systems that require human supervision adding limitations in coverage and time. However, the recent emergence of Wireless Acoustic Sensor Networks (WASN) has allowed non-intrusive remote monitoring of endangered species in real time through the automatic identification of the sound they emit. In this work, an FPGA-based WASN centralized architecture is proposed and validated on a simulated operation environment. The feasibility of the architecture is evaluated in a case study designed to detect the threatened *Botaurus stellaris* among other 19 cohabiting birds species in The Parc Natural dels Aiguamolls de l’Empordà, showing an averaged recognition accuracy of 91% over *2h 55’* of representative data. The FPGA-based feature extraction implementation allows the system to process data from 30 acoustic sensors in real time with an affordable cost. Finally, several open questions derived from this research are discussed to be considered for future works.

## 1. Introduction

Biodiversity describes the full range of different varieties of life on Earth and their diversity. It is a measure a measure of the number and variability of organisms present in different ecosystems. Nevertheless, especially due to climate change, fast environmental changes can cause mass decline or even extinctions of species [1]. The diversity, abundance and identification of animal species have generally been evaluated by different costly methods—in terms of human effort—with limitations in duration and coverage, which usually disregards the generation of permanent data for the subsequent study of time series [2].

The bioacoustic study of specific species and locations may become a useful tool to evaluate the effect of climate change and habitats modification [3,4]. In this context, the acoustic monitoring of birds has been a widely studied area in bioacoustics through the application of sound recognition techniques [5]. However, most of the works found in the literature still follow manual or semi-automatic approaches [6], which still present several open challenges that should be tackled to obtain fully autonomous recognition systems [7], besides making them difficult to scale in wider regions. However, recent technological advances such as Wireless Acoustic Sensor Networks (WASNs) have appeared as an alternative to improve, expand and/or minimize the costs derived from biodiversity monitoring needs around the world [8,9]. For instance, a WASN can be designed and deployed to monitor animal behaviour in a non-intrusive manner, identify several crucial parameters from their habitat—including human interference—or individual counting in a population, among others [9].

Specifically, some bird species have suffered a dramatic reduction of breeding herds in the last decades in the majority of European countries, mainly due to the decrease of the quality and the availability of suitable habitats. They have been catalogued with a high level of protection at community sites by the European Directive 79/409/EEC [10]. In order to inform the competent authorities about the presence of this kind of species in decline in specific habitats and time periods, the development of real-time birdsong recognition system based on the aforementioned WASN paradigm may become of great usefulness [11].

**Figure 1:**
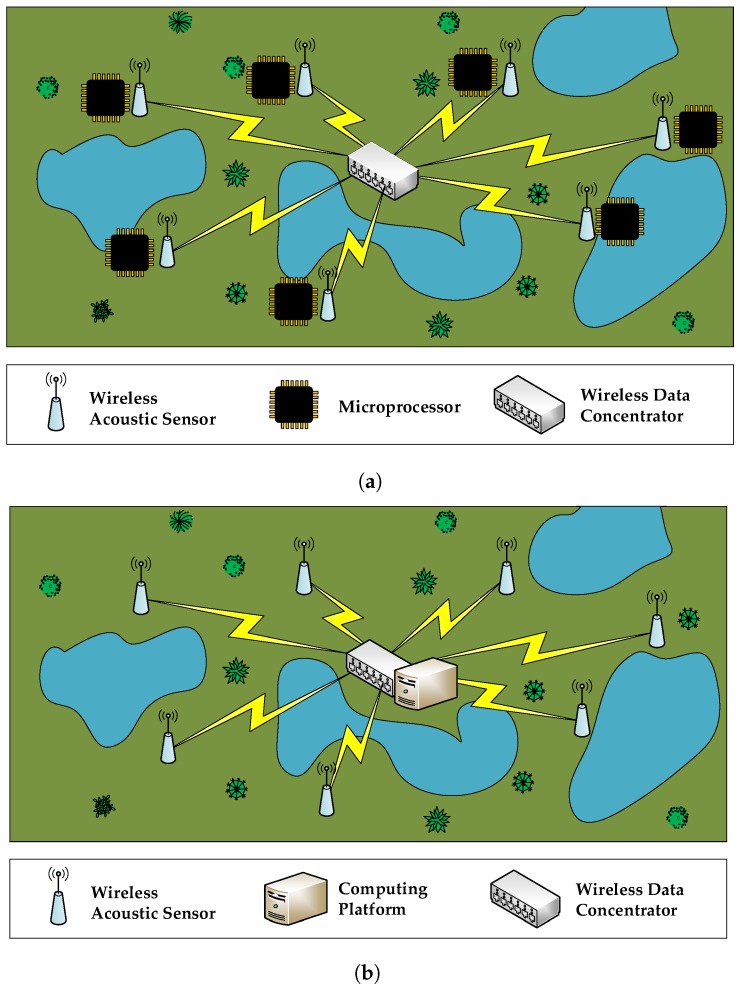
Main alternatives of a wireless acoustic sensor network (WASN) architecture for remote monitoring. (**a**) distributed intelligence; (**b**) centralized intelligence.

When it comes to designing a WASN-based birdsong recognition system to detect the presence of endangered species, two main approaches can be followed. In Figure 1, we can observe the detail of these two proposals: in Figure 1a, the network structure is based on a distributed intelligence, where the nodes have their own computing capabilities to process the raw acoustic data locally, and only transmit to the remote server the results of the evaluations. In Figure 1b, the network structure is based on a centralized intelligence, where the nodes are only used to sense and transmit the raw data to a central system, where they are processed. Those platforms should be low-cost while satisfying the computational requirements. The first approach requires that every node has a computing platform available in order to process the raw data acquired in that node. After the data computation, every node only sends the acoustic event identifier to the central monitoring system. This architecture can be easily scaled since the data communication throughput is low, and the computation is performed in each node independently. Alternatively, the centralized structure is inspired by the Mobile Edge Computing (MEC) paradigm [12], where mobile computing, network control and storage are displaced to the network edges, i.e., base stations and access points. In this case, a core computing platform is needed to process the raw data provided by all the nodes of the network. The key advantage of this latter approach is that network nodes do not need to be provided with high performance computing capabilities. Instead, they can manage the data with a small micro-controller, capable of sensing and performing data transmission. This centralized proposal outperforms the distributed counterpart in terms of overall power consumption and cost of each node.

In the literature, different real-time signal processing platforms can be found, such as Graphics Processing Units (GPUs) [13,14], commercial solutions like Raspberry Pi [15,16] or Field Programmable Gate Arrays (FPGAs) [11]. GPUs present an excellent computational performance to resolve the detection of the threatened species, but they usually have a high price. In addition, we can not guarantee obtaining results in real time due to their timing latency, and their energy consumption is substantially higher than other platforms, which is of paramount interest for wildlife monitoring installations. The Raspberry Pi is a low-cost device, but its performance in terms of computational cost and real-time calculations are not suitable for the problem at hand. FPGAs present a good trade-off between computational capacity with significant low power energy and flexibility at a reasonable cost. Moreover, FPGAs have been already integrated in wireless sensor networks showing satisfactory results, e.g., see [17,18,19]. The reader is referred to [11] for further details about the implementation of sensor nodes relying on FPGAs. Hence, WASN-based wildlife monitoring systems can take advantage of FPGAs to undertake the recognition of the endangered species of interest in real time at a reasonable cost and with low power consumption.

The aim of this work is to design and evaluate the feasibility of an FPGA-based centralized WASN architecture, which includes several acoustic sensors for the remote monitoring of endangered species in real time through the automatic identification of the birdsong in their natural habitat. We validate the architecture by developing a birdsong recognition system in a case study designed to detect the *Botaurus stellaris* songs within a simulated acoustic environment, which includes 19 other bird species cohabiting at The Parc Natural dels Aiguamolls de l’Empordà (Castelló d’Empúries, Spain) [20]. Despite their particular song, the automatic recognition of this species has not been deeply studied yet [21]. Finally, we discuss the key aspects that should be taken into account in order to deploy this kind of architectures in real-life conditions in the future.

This paper is structured as follows. The related work about birdsong recognition, reviewing different FPGA applications in the field of signal processing and the use of WASN, is detailed in Section 2, while the FPGA-based WASN architecture proposal and its validation through a case study designed to detect the threatened *Botaurus stellaris* are found in Section 3 and Section 4, respectively. The discussion of the key aspects of the proposal is detailed in Section 5, and the conclusions and future work can be found in Section 6.

## 2. Related Work

In this section, we revise the main studies performed in automatic birdsong recognition and detail other signal processing applications implemented in real-time over an FPGA. We pay special attention to animal sound recognition and how WASNs have been used as a solution for wildlife monitoring.

### 2.1. Birdsong Recognition

In the literature, we can find several biological projects focused on the acoustic detection of bird songs. The acoustic song recognition is suitable for the monitoring of bird species because many birds are better detected by sound than by vision [6]. In this section, we review some representative works focused on the automatic recognition of bird songs. Depending on the focus of every project or application, the algorithms are designed to identify certain species or to classify diverse species cohabiting in an environment [6]. The statistical machine learning has a central role in the field of bioacoustics and appears in several classification algorithms. After the signal parametrization, the statistical distribution of the audio parameters is modelled using Hidden Markov Models (HMM) [21,22,23], or using the single state version through Gaussian Mixture Models (GMM) [24,25].

In [26], the performance of three different parametric acoustic representations for automatic birdsong recognition is evaluated on 14 common North-European Passerine bird species. The authors include in the study Mel Frequency Cepstral Coefficients (MFCC) [27], due to their good performance in speech recognition, sinusoidal modeling is suitable to represent highly tonal birdsongs and a vector of various descriptive musical-related features because the birdsong can be somehow comparable to music. The best results were obtained by MFCC computed from song segments with no syllable segmentation with respect to the results obtained using syllable segmentation and syllable-specific features.

In [28], each bird sung syllable is segmented into a piece of vocalization, and, for each syllable, the vocalization features are evaluated by means of the averaged Linear Predictive Cepstral Coefficients (LPCC) and the averaged MFCC. Linear Discriminant Analysis (LDA) is used to identify the bird species automatically; the system reaches 87% classification accuracy for a universe of around 420 bird species, covering 561 types of bird songs. In [29], the performance of MFCC is compared with a set of low-level acoustic signal parameters. To that effect, the classification of birdsong is performed by a decision tree with a Support Vector Machine, classifying between two species at each node. The results show that the classification method is invariant to the ordering of the species, and it opens the possibility of weighting the features.

In [30], the detection of spectro-temporal regions that contain the bird vocalizations is based on exploiting the shape of the spectra, identifying sinusoidal components in the short-time spectrum by means of a sine-distance calculation. The bird song identification system uses tonal-based feature extraction and GMM to represent up to 165 bird syllables generated by 95 bird species, obtaining a higher accuracy than using MFCC. In [31], two novel procedures to detect and time-stamp bird songs in continuous real-field recordings are introduced to classify three different bird species. The work is focused on the task of discerning a specific target signal among the rest. In order to confirm the presence of a species in real time, a taxon-specific detector is trained on a large database, which becomes a problem for the amount of data to be processed and labelled.

In [32], the authors detail how features automatically learned from a training dataset can outperform spectrum manually-tuned features, as Mel-spectra feeding the classifier, or any other manually representation to tailor the subject matter. The feature learning stage is conducted in an unsupervised manner, which provides better classification results than MFCC or even Mel-spectra when combined with a *k*-means classifier. Up to twelve different feature extraction coefficients are used to classify four different large databases of bird vocalizations (14,027 audio recordings corresponding to 501 species) by means of random forest. The good performance of the unsupervised approach relies on the database possibilities to train the classification algorithm using only observed data [33].

In [34], an HHM-based system recognizes up to 40 different bird species by means of robust frame selection. The work shows how MFCC offer a good representation of the spectral information for dominant vocalizations because the implementation of a morphological filter eliminates short bursts of noise and weak signals coming from other sources.

### 2.2. Real-Time Automatic Birdsong Recognition

Several approaches to real-time automatic bird recognition can be found in the literature, using hardware platforms covering from microprocessors up to GPUs.

In [16], a Raspberry Pi [35] based platform designed to record acoustic registers is studied. The authors intend to monitor the existence of Hartlaub’s Turaco in central Kenya, which is an endemic species in East Africa that is facing a severe habitat loss due to climate change. After extracting MFCC from the input data, a GMM is applied to classify whether the Hartlaub’s Turaco call is found in the analyzed data or not (i.e., it is a presence/absence classifier). The Hartalaub’s Turaco recognition problem is similar to the *Botaurus stellaris* we are facing in this work, but the proposal does not ensure real-time performance in the sensor. In [15], a prototype for acoustic sensing system named Recording and Observing Bird Identification Node (ROBIN) is developed and deployed in the Cornell Lab of Ornithology (Ithaca, NY, USA). The ROBIN is based on spherical *k*-means to classify the log-scaled Mel-spectrogram representation of each audio clip [36], which outperforms classical MFCC-based approximation [15]. ROBIN works in real-time (based on a Raspberry Pi model B [35]) with a window of 11.6 ms and hops of 1.45 ms.

In [13], the authors focus on reliable detection of bird songs recorded in open field, using the Short Time Fourier Transform (STFT), focusing on its magnitude. The acoustic events appear as blobs, considered the signature of each sound, and Deep Learning is used to classify them. The database is built from the Xeno-Canto Project [37]. The analysis is conducted every 10 ms (with 75% of overlap) in real time using a TITAN-X GPU from Nvidia (Santa Clara, CA, USA) [38], with a time cost of 0.06 s to process around 14 s of data. In [14], the authors try to classify up to 999 different bird species following a similar approach. After processing, the signal in the frequency domain, a convolutional neural network, is applied to train the recognition system over a GPU, allowing real-time performance.

The use of microprocessors, as the aforementioned Raspberry Pi or any other Advanced RISC Machines (ARM) core embedded hardware platforms, present enough potential to address the signal processing and machine learning associated with birdsong classification computational load. However, due to the architecture of the microprocessor, the real-time requirements cannot be guaranteed despite the efficiency of the implemented algorithms. On the other hand, if a GPU hardware is used for single sensor acoustic birdsong detection, most of the approximations are over-dimensioned to achieve real-time performance. Despite its performance as a high computing device, it has high power consumption, which is a clear drawback to work in the wildlife.

#### FPGA-Based Animal Sound Recognition

Three common FPGA signal processing fields of application are speech, music and communications [11]. All of them typically need high speed—usually real-time—complex signal processing algorithms. Nevertheless, FPGAs have also been tested in animal sound recognition environments for their good performance in real-time digital signal processing related tasks, with special attention to their power consumption.

In [39], the authors propose a bird call recognition system based on LPCC using Dynamic Time Warping for a sensor network application. The system is designed to identify two types of birds as a means to validate the proposal: one with a short call length (around 100 ms) and the other with a longer one (around 500 ms). They compare the results obtained using a Xilinx Spartan-3E with a MicroBlaze soft processor (San Jose, CA, USA) with respect to those obtained using an FPGA, with the latter showing about 31.1 times of energy reduction with respect to the MicroBlaze. The authors declare that with a clock of 50 MHz, the power consumption is around 2.8 W.

Finally, in [40], an FPGA-based real-time implementation of an automatic blue whale calls classifier is described. The classification system is based on STFT to extract the acoustic features followed by a multilayer perceptron neural network. Their results are compared to the Matlab (R2012b, MathWorks, Natick, MA, USA, 2012) simulation performance, which uses floating point, while the FPGA implementation is based on a Nexys-4 Artix-7 board (Henley Court Pullman, WA, USA) using fixed point. The authors obtain the same results in Matlab and in the FPGA despite considering different arithmetic, with an averaged recognition accuracy of around 80% for the three classes of whale call.

### 2.3. WASN for Wildlife Monitoring

Wireless Acoustic Sensor Networks have multiple applications, such as surveillance in urban areas, ambient assisted living, environmental or wildlife monitoring [11].

To our knowledge, the first WASN implemented for wildlife monitoring goes back to [41], which worked with a two-tiered sensor network designed to recognize and localize a specific type of bird calls. In [42], the authors detail how self-organizing embedded network sensing technologies can efficiently conduct real-time acoustic source detection and localization by means of embedded devices distributed in the wildlife. In [43], a sensor network is deployed to count endangered bird species in critical environments, helping the census of these threatened species, besides extracting information about their habits. In [44], the authors implemented Kalman filters to track animals, measuring in each sensor node both velocity and acceleration, using a Virtex 4 FX100. The authors partially reconfigure the FPGA in order to reduce the global power consumption, yielding power savings between 5% and 25% in comparison with the fixed implementation.

In [45], a WASN is developed to record audio samples of bird songs in order to study them and extract their fingerprints. The aim of that work is to improve the counting process in a study of endangered animals. They use MFCC to parameterize the input signal, and they classify the birdsong using a J48 decision tree as the machine learning algorithm. Finally, the counting algorithm estimates the number of birds. As far as we know, the project is still under simulation, and it has not been implemented over any hardware platform yet. The work in [46] focuses on the importance of obtaining continuous data of the birds under study in their natural habitat. The work introduces a tonal region detector using a sigmoid function, which gives the system a noise power estimation; once the tonal regions are detected, they extract the features using Gammatone Teager Energy Cepstral Coefficients and introduce them in a classifier based on Deep Neural Networks (DNN). They present results to classify up to 36 bird species from Lake Tonga.

Finally, it is worth noting that most of the applications found in the literature apply the signal processing algorithms over the raw data in each node of the network as they are based on a distributed architecture, which increases the total cost of the network design.

## 3. An FPGA-Based WASN Centralized Architecture for Real-Time Remote Monitoring of Endangered Species

We present a WASN system following a centralized architecture paradigm based on a Zynq device (Xilinx Inc., San Jose, CA, USA). This centralized architecture, where the data processing takes place in a single device, allows us to implement simpler nodes than those presented in the related work, where a microprocessor or a FPGA are used in every single node to perform the signal processing. This simplification in the nodes reduces their costs and power consumption, which are the main constraints in WASNs deployed in the wild.

**Figure 2:**
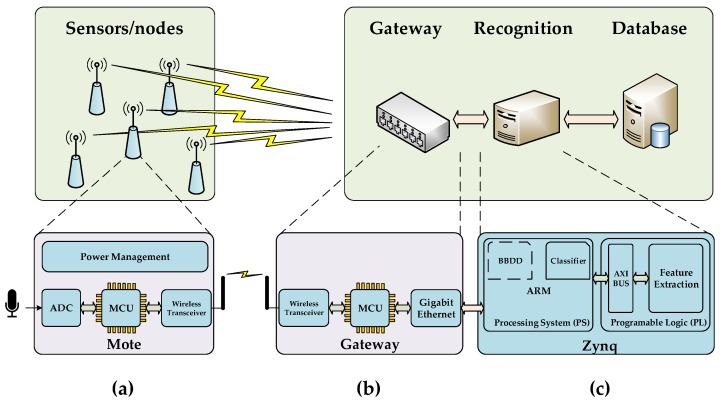
System proposal detailed where the three basic elements are presented: (**a**) node; (**b**) gateway; and (**c**) Field Programmable Gate Array (FPGA)-based centralized computing platform based on a Zynq.

The proposed architecture to design an FPGA-based centralized WASN is depicted in Figure 2. This system contains three main elements: (*a*) simple nodes or motes, which are in charge of sensing the environment sounds and sending the raw data through wireless communication to the gateway, (*b*) the gateway, which is a subsystem able to coordinate the wireless network and collect all the raw data from the nodes re-transmitting them to the centralized recognition system, and (*c*) the recognition system, which provides the machine hearing functionality through the application of audio input feature extraction and machine learning. Moreover, a database to store the results of the analysis can be used remotely or included it in the recognition system platform.

The proposed hardware platform is based on a System on Chip (SoC) built on a Zynq FPGA. This kind of platform offers the possibility to implement algorithms of high computational cost in the programmable logic (PL) of the FPGA, which is a suitable solution when execution time is an important constraint. With such platform, while the FPGA implements the algorithms, the processing system (PS) of the ARM would implement an operating system to control the applications and the systems peripherals easily.

Currently, Zynq devices allow high granularity between the low cost and the high performance fabric, as we can see in Table 1, where the highest performance device has more than ten times the resources of the lowest cost version. These devices will be taken into account in the available computational resources analysis.

The FPGA contains the aforementioned bird recognition algorithm encapsulated as a whole intellectual property (IP) with Advanced Micro-controller Bus Architecture (AMBA) Advanced eXtensible Interface (AXI4) interface to communicate with an ARM microprocessor, which will be responsible for the control of the FPGA and which will include the machine learning classifier as well. AXI4 buses were adopted by Xilinx and are widely used to easily connect IPs with embedded micro-controllers or ARM cortex-A microprocessors such as MicroBlaze or Zynq, respectively, with several peripherals [48]. Figure 2 shows a block diagram of the proposed connectivity between modules or entities for the whole system, where the proposed IP calculates efficiently the feature extraction of the measured acoustic signal, and the ARM embedded in the Zynq platform conducts non-intensive tasks such as the network management and the automatic classification.

As stated before, the interfaces of this IP are based on AXI4 buses, which allow for establishing handshake between modules to read and write information with the aim of avoiding information loss due to low capacity buffers in some components. Both input and output interfaces have an asynchronous First Input First Output (FIFO) memory, using on one side the IP system clock and on the other side the ARM clock, since those might be the same speed or not, and might be synchronous with each other or not. This commonly used technique allows not only two clock domains running at different speeds (which is very common in SoCs), but also minimizes data loss and makes the system more agile since the FIFO can save as many data words from the fast clock as its depth.

## 4. System Validation: Birdsong Recognition of *Botaurus stellaris* among Cohabiting Species

In order to validate the proposed system architecture, we develop a case study focused on the detection of *Botaurus stellaris* birdsong among cohabiting species in a simulated environment. This is a key step before deploying an FPGA-based WASN designed to detect this species in protected places where their presence for reproduction is unknown nowadays. The common bittern (*Botaurus stellaris*), whose loud booming call performed by males during the breeding season can be heard from up to 2 km away [49,50], is nowadays catalogued with a high level of protection at Community-(Castelló d’Empúries, Spain) [20].

**Figure 3:**
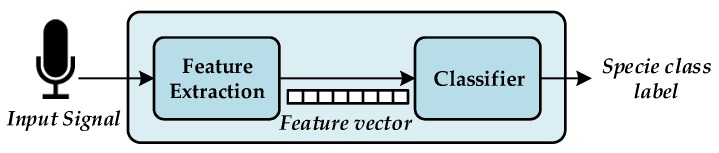
Block diagram of the birdsong recognition system where the main elements are depicted: (**a**) the feature extraction obtains a representation with lower dimensionality of the input signal with the aim of characterize it and (**b**) the classifier lebels the features extraction frames with a specie class.

The acoustic birdsong recognition algorithm implemented by the WASN consists of two different stages, as detailed in Figure 3: feature extraction and classification. Feature extraction is a signal processing procedure that aims to parametrize the key characteristics of the audio birdsongs by means of a set of representative coefficients with a lower dimensionality than the original samples [51]. Once these coefficients are available, they are input into the classification module, which is asked to identify the birdsong class from those learned from the training stage. To that effect, the classifier is trained using a representative labelled dataset.

We select MFCC [27] as the feature extraction technique based on the perceptual Mel scale [52], since it has been explicitly used or included as the baseline in most works focused on the detection of animal sounds, being a good representation of this kind of information [34]. Moreover, we consider GMM [34] as a representative machine learning approach within this research field, besides providing good results in similar experiments [24,25]. The birdsong recognition algorithm has been developed and tested in a Matlab environment with a synthetic dataset in order to validate the accuracy of the system.

### 4.1. Dataset Description

The dataset used to train and test the developed system includes the *Botaurus stellaris* and 19 coexisting bird species in The Parc Natural dels Aiguamolls de l’Empordà. The dataset lasts 8 h and 18 min approximately, with 30 recording files per species collected from the Xeno-Canto project [37] sampled at 48 kHz. The duration of the recording for each species of the dataset is detailed in Table 2. The audio recordings contain bird songs and silences; thus, a pre-processing stage should be conducted to annotate the corpus, annotating the birdsongs and the silence intervals. To this aim, we have opted for a straightforward approach by selecting the 35% highest energy frames of each file as a birdsong, thus discarding the remaining frames, which entails reducing the total duration of the corpus from 8 h and 18 min to 2 h and 55 min. Following this approach, we have assured that we are only considering birdsongs for the case study, in addition to avoiding the effort to generate a manually revised dataset—a process that is left for future work on real-life collected data.

**Figure 4:**
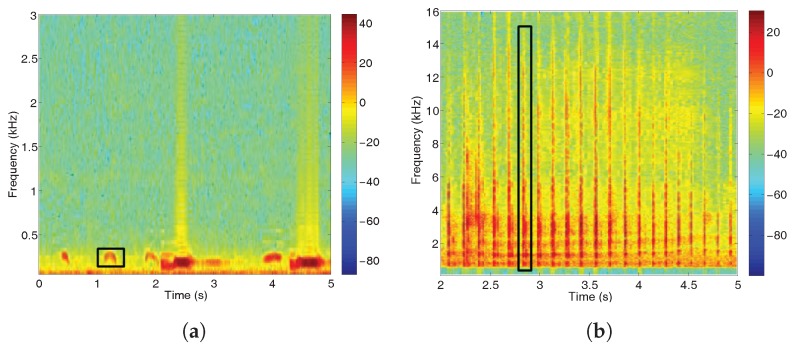

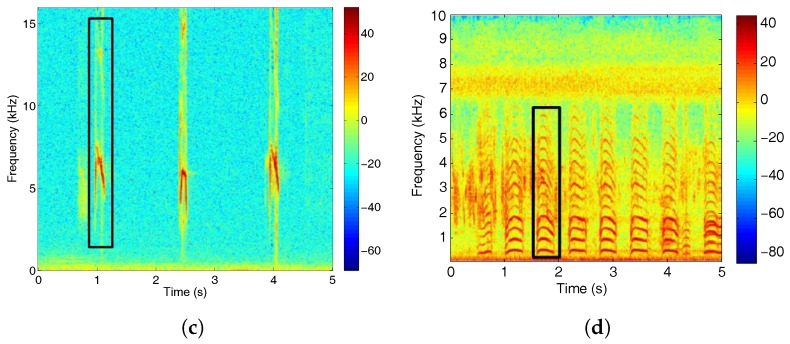
Spectrograms for several birdsongs where call examples have been highlighted. (**a**) *Botaurus stellaris*; (**b**) *Ciconia ciconia*; (**c**) *Motacilla flava*; and (**d**) *Porphyrio porphyrio*.

The call of the *Botaurus stellaris* is mainly characterized by its center frequency around 150 Hz, which can be detected thanks to the energy peaks in the suitable frequency band in low noise conditions [21]. Figure 4a shows the spectrogram of a *Botaurus stellaris* call, while Figure 4b–d show the spectrogram of a *Ciconia ciconia*, *Motacilla flava* and *Porphyrio porphyrio*, respectively. These spectrograms reveal notable acoustic and spectral differences between those species, as the bittern presents a narrow-band signal centered at very low frequencies, whilst *Ciconia ciconia*, *Motacilla flava* and *Porphyrio porphyrio* calls cover a wider spectrum at different higher frequencies up to 15 kHz, as it can be observed in Figure 4b,c.

### 4.2. Feature Extraction

**Figure 5:**
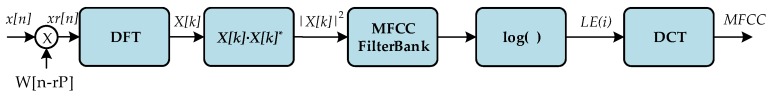
Bloc diagram of the Mel Frequency Cepstral Coefficients (MFCC) extraction procedure from the input acoustic signal x(n), where W[n−rP] is the Hamming windowing, X[k] is the discrete Fourier transform of the framed input signal, LE(i) is the logarithm of the filter bank coefficients and MFCC the output array.

The process followed to compute MFCC is shown in Figure 5, where the incoming audio signal is divided into frames of 30 ms, which correspond with 1440 samples with 50% overlapping to compensate the power reduction in the tails of the window to avoid losing audio events in these time slots. These frames are transformed to the frequency domain using a Discrete Frequency Transform to evaluate the contribution of every band of the spectrum. This step uses a filter-bank of 48 filters, distributed from 20 to 1000 Hz, whilst the rest arrives up to 16,000 Hz following the Mel-scale. The final coefficients are obtained after extracting the first 13 coefficients of the Discrete Cosine Transform (DCT). The high order MFCC are discarded to reduce the parametrization dimensionality. This can be done due to the fact that the main information of the acoustic events can be found in the lower DCT region, which corresponds to the signal envelope [27]. As a result, each 30 ms audio signal frame is characterized with a vector of 13 components, which is fed to the classifier.

The proposed implementation of the MFCC algorithm in the FPGA described in Appendix A is summarized in terms of the consumed resources in Table 3, which shows the resource consumption of each main component separately, as well as the total consumption of our IP once packaged. The execution time has also been calculated in number of cycles using the Xilinx IP cores’ information and our Finite State Machines (FSM) designs. Please note that, in order to get detailed information about each block, they had to be implemented separately, which means the Place and Route tools of the software Vivado (2016.7, Xilinx Inc., San Jose, CA, USA) might have been optimized differently than when the whole IP was present.

From Table 3, we can observe that the most restrictive component of the designed algorithm in terms of execution time is the MFCC_FSM block, which requires 49,156 rising clock edges to process a new audio frame. This means that, with a clock frequency of 100 MHz, the proposed algorithm would process a whole frame of 30 ms of audio in 500 μs approximately, which is 49,156 rising clock edge at 10 ns. Therefore, the number of sensors or independent audio frames that can be processed in real time, considering a 50% of analysis overlapping, is 30 because 30×500μs is 15 ms.

In Table 4, we show the resource consumption of our IP as a % of the total available resources in the four lower resources of Zynq devices described previously. Please note that for the implementation in Vivado we defined the device as an XC7Z020; therefore, if we would use another device, the Place and Route tool would likely optimize differently and give slightly different results. Despite this, with the post-implementation resource consumption calculated by Vivado, we can see how our IP would easily fit in any device bigger than the XC7Z010 and still have most of the resources available. In case we decided to use a XC7Z010, optimization of blocks like the MFCC_FSM could be used prior to implementation to reduce the amount of Block RAM required, which would require a higher use of other resources but would make this device suitable for the application.

### 4.3. Machine Learning for Audio Birdsong Recognition

The audio birdsong recognition module aims to label automatically the correct species by means of the characterized MFCC corresponding to the 30 ms audio frames. For this purpose, this algorithm takes as an input the aforementioned 13-dimension vector from the feature extraction module obtaining as output the inferred label of the recognized species, considering an automatic classifier with supervised learning.

**Figure 6:**
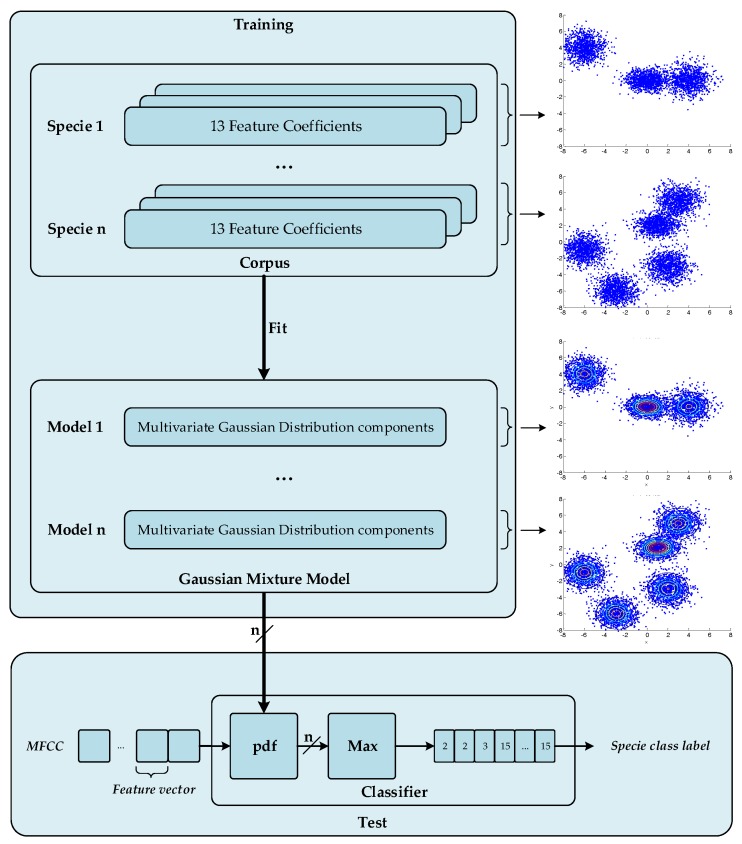
Training and test procedures of the proposed audio birdsong recognition.

The supervised machine learning algorithm used is based on a GMM-approach [53] using spectral shape features; both (a) training and (b) test procedures are depicted in Figure 6. First of all, the training data is segregated into *n* different sets of MFCC vectors corresponding to the *n* classes or species that we aim to classify. The k-means algorithm, which is an unsupervised learning algorithm, performs the estimation of the mean of every cluster for the *n* classes, which are modeled as a combination of different clusters of data. Subsequently, the mean of every cluster and the total number of clusters are fed the GMM fitting algorithm with the aim to approximate the cloud of data with a combination of multivariate Gaussians. We have conducted a comparative study considering a sweep of the number of mixtures. As you can see from Appendix B, M=32 represents a good trade-off between classification accuracy and computational cost, as already observed in [21]. Finally, we obtain n=20 Gaussian Mixture Models of M=32 multivariate Gaussians with d=13 components. Once the system has been trained with *n* GMM models, the test data set is processed calculating the highest probability density for the *n* GMM inferring the class label for these audio frames.

The aforementioned GMM for multi-class supervised classification uses *n* models based on *M* multivariate Gaussian Distributions of dimension *d* for every model to obtain the class with the highest probability. The probability of every model $\left(P_{i}\left(x,\theta_{i}\right)\right)$ is obtained as a linear combination of *M* Gaussian distributions and a weights function $w_{k}$, as described in Equation (1). The computation of every multivariate Gaussian distribution is obtained from Equation (2), where $\Sigma_{k}$ represents the co-variance matrix:

(1) $$P_{i}\left(x;\theta_{i}\right)=\sum_{k=1}^{M}p_{k}\left(x;\mu_{k},\Sigma_{k}\right)w_{k},\;\theta_{i}=\left\{\mu_{i,1},w_{i,1},\Sigma_{i,1},...,\mu_{i,d},w_{i,d},\Sigma_{i,d}\right\},\;w_{k}\geq 0,\;\sum_{k=1}^{M}w_{k}=1,$$

(2) $$p_{k}\left(x;\mu_{k},\Sigma_{k}\right)=\frac{1}{{\left(2\pi \right)}^{\frac{d}{2}}{\left|\Sigma_{k}\right|}^{\frac{1}{2}}}exp\left[-\frac{1}{2}{\left(x-\mu_{k}\right)}^{T}\Sigma_{k}^{-1}\left(x-\mu_{k}\right)\right].$$

Therefore, the implementation of the GMM algorithm requires obtaining n·M multivariate Gaussian distributions of dimension *d*. The first part of Equation (2), $|\Sigma_{k}|$ and $\Sigma_{k}^{-1}$ are constant values and arrays that can be pre-calculated for each of the n·M multivariate Gaussian distributions with no contribution to the computational cost of the algorithm at run time. The computational cost for the proposed GMM algorithm with 20 classes, 32 mixtures and 13 dimensions is 225,920 floating point operations per frame, as detailed in Table 5. Finally, the total computational cost is 451,840,000 floating point operations per second (FLOPS), since the audio frame rate is set to 15 ms and the number of wireless remote sensors is 30. The NEON, which is a single instruction-multiple data (SIMD) engine provided with every ARM Cortex-A9 of the Zynq platform, is a co-processing unit that can deal with this computational cost. In fact, running at 1 GHz, it can achieve a maximum of 2 GFLOPS [54]. For this reason, this algorithm can be executed in the PS of Zynq devices. However, it can be executed partially in the PL to accelerate it. Table 5 shows that the performance of the GMM in terms of computational depends linearly on the number of mixtures (M) and the number of classes (n). Thus, we can multiply the number of classes or the number of mixtures by up to four, and despite this increase of computational load, the PS microprocessor, jointly with the NEON, should be able to process the GMM.

### 4.4. Performance of the Birdsong Recognition

**Figure 7:**
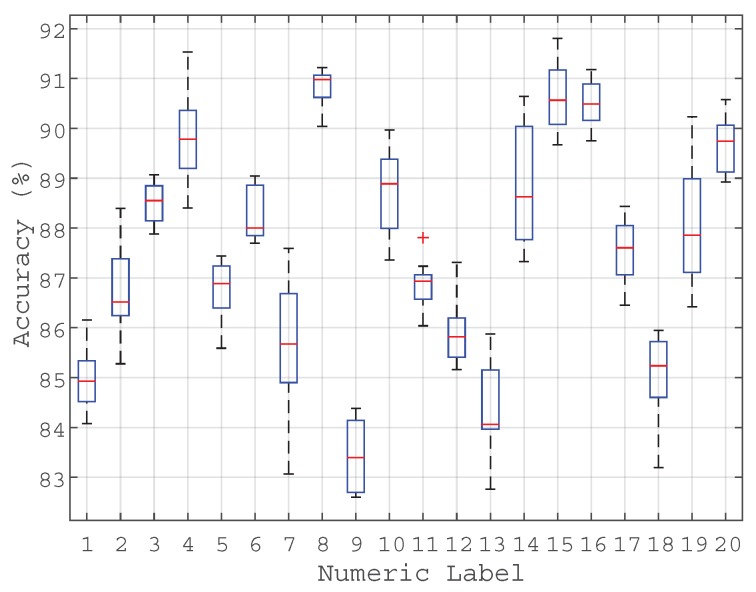
Accuracy boxplots of the birdsong classifier obtained from the 10-fold cross-validation. The numerical labels correspond to the species listed in Table 2.

The developed birdsong recognition system has been evaluated following a 10-fold cross-validation scheme [55], which means that the classifier has been trained using 90% of the audio samples and tested with the remaining 10% of data at each fold. The results in terms of the system’s classification accuracy are depicted in Figure 7 and Table 6, showing the boxplots of the results from the 10-fold cross-validation and the confusion matrix in terms of mean accuracy, respectively.

From Figure 7, we can observe that the achieved accuracy presents averaged values ranging from 82% to 92% per bird species, whilst the accuracy variance is lower than 2%.

We can also observe that the *Botaurus stellaris*, which is the target endangered species under study, has been classified with a mean accuracy of 90.9% (labelled as species number 8 in Figure 7), which is among the best results of the experiment. After an initial inspection of the diversity of bird songs under classification, this can be probably due to the singularity of its song. However, this hypothesis should be confirmed through specific analyses in future works.

## 5. Discussion

In this section, we discuss several open questions derived from the proposal and the case study developed in this work. We have taken into account the real-time implementation of the described birdsong recognition system, and several key issues related to signal processing and the improvement of the machine learning algorithm, as well as ideas to take into account for the FPGA-based WASN architecture for its future implementation.

### 5.1. Remote Wireless Acoustic Sensors: Motes

The proposed centralized architecture is based on several simple motes with the functionality of sampling the environment sound and transmitting it to a wireless gateway to be processed in a centralized platform. This approach offers the following benefits with respect to a distributed architecture: (a) the power consumption and (b) the cost of the nodes can be lower because each can be implemented with a single micro-controller instead of applying the signal processing algorithm over the raw data in each node using a microprocessor or FPGAs that has been implemented previously in a distributed architecture in the literature, due to the fact that the digital signal processing can be computed in the embedded platform. This reduction in the power consumption, which means an increase of the battery life and in the total cost, are one of the main constraints in WASN deployed in the wild.

Future work should focus on the study and comparison of the different available wireless technologies with the aim to increase the battery life. Moreover, some techniques based on the sound event detection can be implemented to choose which audio frames should be transmitted or not to the centralized platform to reduce the transmission time. Despite the fact that it would reduce the power consumption of the system, it can only be applied if the micro-controller is able to assume the computational cost of the algorithm.

Finally, the coverage area of the whole system depends on microphone directivity, wireless technology coverage and the maximum number of nodes gathered by the WASN.

### 5.2. Platform Proposal for the Centralized Architecture

Based on our preliminary implementation, we propose a set of low cost platforms suitable to be used in the deployment of the whole system in the future. Some of them are listed in Table 7. They are provided with the XC7Z010, XC7Z015, XC7Z020 or XC7Z030 device; as was stated previously, XC7Z015, XC7Z020 and XC7Z030 have more available resources than the algorithm requirements (see Table 4). In the case of working with an XC7Z010, resource optimization should be applied to the algorithm prior to implementation in order to reduce the amount of Block RAM consumed.

#### Real-Time FPGA Performance

The presented birdsong recognition system is made up of a feature extraction which has been developed on the PL side of the Zynq, and a machine learning classifier which has been developed in the PS side. With the resources required in the feature extraction implementation on the PL side, which is detailed in Section 4.2, we can conclude that up to approximately 30 sensors could be processed in real-time within the same FPGA, providing the upper bound of our WASN design; despite this, the FFT implementation (Radix-2 Lite Burst Input/Output (I/O) (Xilinx Inc., San Jose, CA, USA) could be an item to study so as to optimize its performance [56]. Moreover, the serial implementation of the MFCC_FSM, which is the most restrictive component of the process in terms of execution time, presents high memory consumption, and, despite using few DSPs and LUTs, it could be implemented in parallel, decreasing the execution time at the same time that it increases the number of used DSPs and LUTs. Apart from that, the implemented machine learning algorithm based on GMM has a computational cost, which is linearly dependent on the number of classes and the number of mixtures. Then, we could increase one of these features up to four times, allowing this algorithm to be fully implemented on the PS side of the Zynq.

Finally, if the number of sensors to monitor was smaller than 30, the frequency clock of the system could be lower than the proposed 100 MHz to drastically reduce the power consumption because the dissipation is a function of the system frequency [57]. The fine tuning of the proposed algorithm should be done depending on the time execution, the area and power constraints of the application.

### 5.3. *Botaurus stellaris* Song Identification

As a case study, the developed bird song recognition system shows good results when tested in a simulated environment over Matlab. In particular, the *Botaurus stellaris* recognition accuracies are placed among the best ones. Despite MFCC presenting good results for the classification conducted in this paper, other feature extraction techniques could be considered in the future, especially those specifically designed to represent music and songs [51].

This work has been conducted considering a typical configuration using MFCC and GMM and a synthetic dataset, with the aim to validate the whole proposal, leaving for future works its optimization. Although we have validated that working with 32 mixtures allows a good trade-off between recognition accuracy and computational cost, it is worth mentioning that, in our experience in similar investigations [58,59], we have observed that configurations that perform optimally in synthetic datasets may not correspond to the best approaches to deal with real-life recorded data. Therefore, the optimization of the feature extraction and the machine learning algorithms should be conducted again with real-life recorded data.

For instance, in the feature extraction stage, not only the spectral information could be taken into account, but also the temporal information [6]. Due to the particularities of the *Botaurus stellaris* song, a spectro-temporal analysis would likely improve the classification results because the temporal information in terms of repetitiveness, combined with the spectral occupation (closer to lower frequencies than nearly any other species), could improve the separability of our target endangered species.

### 5.4. Classification Approaches

Gaussian Mixture Models have good overall performance in the tests conducted in this paper; despite this fact, there is some room for improving the robustness of the system to identify of the *Botaurus stellaris* among coexisting bird species before moving the automatic recognition system from the simulated to the real-life environment. For that purpose, the authors consider that one of the potential next steps should compare the GMM performance to other machine learning algorithms. Multiple stage machine learning algorithms such as HMM [21,22] can be tested in this environment as an extension of the preliminary results presented in this piece of research.

According to several authors, Deep Neural Networks show good performance in automatic birdsong recognition, improving classical machine learning methods substantially when enough training data is available. In [60], the technical program of LIFEClef 2016 describes the future of birdsong recognition centered in DNN, as, for instance, the one described in [61]. The strong disadvantage of the use of DNN is the huge amount of annotated data needed to train suitably the NN, which, specially in the case of endangered species, may become an almost unfeasible challenge if the acoustic data should be collected from real-life scenarios.

Another approach worth investigating is the consideration of the target bird to be identified in comparison to the soundscape of interest and all cohabiting birdsongs. This approach would convert our *n*-classification problem (in our case, n=20) into a one-type identification [16] or a binary classification problem [58]; we should explore whether these two approaches would be useful to improve the obtained results with the synthetic dataset—as done in this work—but especially analyze their performance in real-life recordings, with species song overlap and environmental noise.

## 6. Conclusions

This work has proposed a centralized Wireless Acoustic Sensor Network based on a Zynq Xilinx FPGA, which is the device that conducts the real-time digital signal processing, with the aim to detect endangered bird species in specific habitats such as natural parks. The centralized architecture allows non-intrusive remote monitoring of endangered species emitting specific sounds in real time by using very simple micro-controllers to develop the sensor nodes, which reduce the power consumption and cost of these platforms.

The aforementioned proposal has been validated through a case study designed to detect the *Botaurus stellaris*—which is species of The Parc Natural dels Aiguamolls de l’Empordà in severe recession—in a simulated environment by using a synthetic dataset generated from the Xeno-Canto project, MFCC to parameterize the input audio data and GMMs to model them as machine learning algorithms. The results demonstrate the viability of the proposal, showing an overall recognition accuracy of the bird species between 82% and 92%, after following a 10-fold cross-validation scheme run on the *2h 55’* dataset. Specifically, the *Botaurus stellaris* presents one of the best accuracy distributions (with a mean accuracy of around 91%) when identified among the remaining 19 cohabiting bird species’ songs.

This work has also detailed the MFCC implementation on an FPGA device using a Zynq platform in which an ARM cortex-A and an FPGA coexist. Finally, an analysis of low cost Zynq platforms is done based on the required resources of the proposed MFCC plus GMM algorithm, concluding that up to 30 acoustic sensors can be processed in real time within the same FPGA as the upper bound of the WASN centralized architecture.

In terms of the implementation of the birdsong recognition system, we have observed that the Mel scale filter bank entails the highest execution cost. Since the series implementation still shows acceptable results in terms of time consumption (i.e., it allows our IP to process data in real-time), we have opted for this implementation focusing on the reduction of the consumed resources (DSPs and LUTs) instead of minimizing the execution time (i.e., using a parallel implementation). This is possible due to the low throughput of the input data. However, some extra optimization can be addressed in the future by increasing the degree of parallelization as well as choosing a more intensive architecture implementation in the FFT IP core. Both approaches would allow for processing hundreds of sensors at the cost of increasing the amount of computational resources required (e.g., Block RAMs, DSPs, FFs and LUTs). These low-level adjustments should be performed depending on the optimization required by the specific application, the most important parameters being the number of sensors, the latency and execution time, and the computational resources and the power consumption.

After this research focusing on the validation of the FPGA-based WASN architecture by means of a specific case study, we plan to design and develop a complete WASN-based system composed of several low-power microphones integrating the communication infrastructure in a real-life environment. These devices should have very low power consumption in order to extend lifespan of their powering batteries. Their main function will be to gather the acoustic data and send it to the centralized module through the gateway, namely, the aforementioned Zynq FPGA of Xilinx (or some of the equivalent platforms referred to in the paper). Nevertheless, the specific requirements and design for the whole system (e.g., number of devices, location, connectivity, etc.) should be studied in detail before its implementation in future works. Moreover, the specific study about the geographic area coverage of the designed and validated WASN architecture in The Parc Natural dels Aiguamolls de l’Empordà is left for future works.

Finally, it is worth noting that this work has been done in a simulated environement using a synthetic database. Therefore, a complete data set collected from The Parc Natural dels Aiguamolls de l’Empordà should be obtained and annotated accurately to evaluate the implemented system in real-life conditions with the same device characteristics. To that effect, a recording campaign should be conducted during several days/weeks in different places of the park in order to obtain enough representative environmental sounds and birdsongs in an open field. Nevertheless, one of the key issues of the recordings will be related to the collection of *Botaurus stellaris* songs, which remains as an open question at the current stage of our research, since it is a species in severe recession in that natural park.

## Figures and Tables

**Table 1:** Zynq-7000 family overview [47].

Part Number	XC7Z010	XC7Z015	XC7Z020	XC7Z030	XC7Z035	XC7Z045	XC7Z100
Look-Up Tables	17,600	46,200	53,200	78,600	171,900	218,600	277,400
Flip-Flops	35,200	92,400	106,400	157,200	343,800	437,200	554,800
Block RAM (Mb)	2.1	3.3	4.9	9.3	17.6	19.1	26.5
(# 36 Kb Blocks)	(60)	(95)	(140)	(265)	(500)	(545)	(755)
DSP Slices	80	160	220	400	900	900	2020

**Table 2:** Total duration of the data set used with the most populated species in The Parc Natural dels Aiguamolls de l’Empordà.

Numeric Label	Species	Duration Min(${}^{\prime }$) Sec(${}^{\prime \prime }$)
1	*Acrocephalus arundinaceus*	37${}^{\prime }$15${}^{\prime \prime }$
2	*Acrocephalus melanopogon*	21${}^{\prime }$57${}^{\prime \prime }$
3	*Acrocephalus scirpaceus*	47${}^{\prime }$11${}^{\prime \prime }$
4	*Alcedo atthis*	15${}^{\prime }$1${}^{\prime \prime }$
5	*Anas platyrhynchos*	22${}^{\prime }$4${}^{\prime \prime }$
6	*Anas strepera*	18${}^{\prime }$27${}^{\prime \prime }$
7	*Ardea purpurea*	11${}^{\prime }$38${}^{\prime \prime }$
8	*Botaurus stellaris*	25${}^{\prime }$20${}^{\prime \prime }$
9	*Charadrius alexandrinus*	18${}^{\prime }$28${}^{\prime \prime }$
10	*Ciconia ciconia*	17${}^{\prime }$15${}^{\prime \prime }$
11	*Circus aeruginosus*	23${}^{\prime }$11${}^{\prime \prime }$
12	*Coracias garrulus*	45${}^{\prime }$50${}^{\prime \prime }$
13	*Dendrocopos minor*	26${}^{\prime }$5${}^{\prime \prime }$
14	*Fulica atra*	8${}^{\prime }$36${}^{\prime \prime }$
15	*Gallinula chloropus*	23${}^{\prime }$10${}^{\prime \prime }$
16	*Himantopus himantopus*	51${}^{\prime }$7${}^{\prime \prime }$
17	*Ixobrychus minutus*	29${}^{\prime }$43${}^{\prime \prime }$
18	*Motacilla flava*	26${}^{\prime }$1${}^{\prime \prime }$
19	*Porphyrio porphyrio*	11${}^{\prime }$24${}^{\prime \prime }$
20	*Tachybaptus ruficollis*	18${}^{\prime }$27${}^{\prime \prime }$
	***Total***	8 h 18${}^{\prime }$10${}^{\prime \prime }$

**Table 3:** Execution time and required resources for the full implementation proposed of the Mel Frequency Cepstral Coefficients (MFCC) algorithm.

Component	Execution Time	Required Resources			
	(# of Clocks)	DSPs	Block RAMs	LUTs	FFs
2xFIFOs Depth 2048	–	0	4	218	0
FFT_FSM	28,713	23	7	1551	1933
RAM	–	0	1	0	0
ROM	–	0	64	32	1
MFCC_FSM	49,156	2	0	773	1263
LOG	–	4	0	710	1192
DCT_FSM	53	2	1	1254	2987
**TOTAL**	–	**31**	**73**	**4489**	**7677**

**Table 4:** Resources consumed by our IP compared to the available resources in Zynq devices.

Device	Required Resources in %			
	DSPs	Block RAMs	LUTs	FFs
XC7Z010	38.75	**121.66**	25.5	21.8
XC7Z015	19.37	76.84	9.7	8.3
XC7Z020	14.09	52.14	8.4	7.2
XC7Z030	7.75	27.5	5.7	4.9

**Table 5:** Computational cost of the Gaussian Mixture Models (GMM) algorithm for the evaluation of *n* probabilities of each frame of 30 ms, where *n* is the number of classes, *M* the number of Gaussians and *d* the dimension of the features.

Operation	Computational Cost	Floating Point Operations
Subtractions	n·M·d	8320
Multiplications	$n\cdot M\cdot (d^{2}+d+1)$	117,120
Additions	$n\cdot M\cdot ({\left(d-1\right)}^{2}+d)$	100,480
**TOTAL**	–	**225,920**

**Table 6:** Confusion matrix of the GMM-based birdsong classifier. Predicted classes are on the columns and actual classes are on the rows.

	1	2	3	4	5	6	7	8	9	10	11	12	13	14	15	16	17	18	19	20
1. *Acrocephalus arundinaceus*	84.97	1.44	2.28	0.69	0.71	0.63	0.62	0.26	0.65	0.55	0.96	1.31	0.59	0.24	0.65	0.28	0.35	1.07	0.68	1.07
2. *Acrocephalus melanopogon*	1.20	86.75	2.74	0.63	0.42	0.39	0.48	0.41	0.81	0.43	1.01	0.45	0.62	0.17	0.28	0.28	0.66	1.23	0.40	0.63
3. *Acrocephalus scirpaceus*	1.48	1.99	88.50	0.35	0.18	0.41	0.22	0.42	0.71	0.37	0.94	0.47	0.63	0.21	0.33	0.51	0.57	0.75	0.42	0.53
4. *Alcedo atthis*	0.45	0.58	0.22	89.87	0.40	0.18	0.43	0.75	0.41	0.15	0.76	0.32	1.12	0.75	0.90	0.10	0.72	1.48	0.21	0.18
5. *Anas platyrhynchos*	0.89	0.42	0.30	0.31	86.79	1.82	0.93	0.27	0.39	1.16	1.02	0.97	0.73	0.59	0.78	0.10	0.46	0.47	1.10	0.50
6. *Anas strepera*	0.58	0.68	0.36	0.19	2.13	88.23	0.75	0.55	0.30	0.82	1.18	0.78	0.39	0.38	0.35	0.08	0.53	0.25	1.01	0.46
7. *Ardea purpurea*	0.53	0.39	0.34	0.60	0.96	0.94	85.65	0.35	0.87	0.89	0.89	0.51	0.76	0.47	0.56	1.16	0.90	1.76	0.62	0.85
8. *Botaurus stellaris*	0.40	0.53	0.29	0.40	0.23	0.32	0.25	90.85	1.40	0.38	0.75	0.69	0.63	0.82	0.20	0.15	0.80	0.39	0.34	0.17
9. *Charadrius alexandrinus*	0.58	0.51	0.60	0.53	0.36	0.21	0.66	1.02	83.45	0.11	0.58	1.66	0.59	1.29	0.24	3.68	0.46	2.79	0.16	0.53
10. *Ciconia ciconia*	0.54	0.50	0.60	0.24	1.14	1.30	0.69	0.42	0.37	88.72	0.67	0.81	0.40	0.75	0.49	0.20	0.71	0.25	0.88	0.34
11. *Circus aeruginosus*	0.62	0.92	0.72	0.78	0.80	0.68	0.66	0.56	0.36	0.45	86.90	0.75	0.94	0.76	0.59	0.47	0.62	0.45	0.83	1.13
12. *Coracias garrulus*	1.20	0.48	0.48	0.38	1.02	0.57	0.45	1.16	1.99	0.69	0.74	85.98	0.83	0.39	0.47	0.47	0.65	0.87	0.42	0.77
13. *Dendrocopos minor*	0.59	0.50	0.62	1.38	0.81	0.49	0.61	0.76	0.67	0.51	1.15	0.60	84.39	1.02	0.47	2.25	1.45	0.95	0.32	0.46
14. *Fulica atra*	0.40	0.18	0.16	1.06	0.96	0.54	0.35	0.52	0.99	0.58	0.81	0.33	0.75	88.87	0.83	0.21	0.76	0.35	0.65	0.70
15. *Gallinula chloropus*	0.61	0.34	0.45	0.52	0.79	0.34	0.52	0.20	0.31	0.32	0.77	0.57	0.67	0.57	90.66	0.26	0.57	0.18	0.74	0.60
16. *Himantopus himantopus*	0.28	0.32	0.34	0.10	0.09	0.08	0.45	0.15	1.73	0.08	0.40	0.32	0.78	0.11	0.27	90.51	0.45	3.08	0.15	0.31
17. *Ixobrychus minutus*	0.39	0.67	0.49	1.05	0.72	0.50	0.66	0.76	0.53	0.72	0.67	0.77	1.23	0.65	0.53	0.57	87.49	0.34	0.89	0.38
18. *Motacilla flava*	0.62	1.03	0.81	1.59	0.44	0.21	1.10	0.32	2.49	0.19	0.37	0.62	0.86	0.22	0.19	3.44	0.25	85.01	0.14	0.12
19. *Porphyrio porphyrio*	0.66	0.47	0.53	0.15	1.61	1.01	0.48	0.76	0.16	0.70	1.07	0.49	0.42	0.64	0.65	0.11	0.89	0.35	88.06	0.79
20. *Tachybaptus ruficollis*	1.07	0.71	0.55	0.21	0.55	0.36	0.67	0.12	0.56	0.46	1.05	1.04	0.43	0.49	0.57	0.46	0.42	0.19	0.41	89.69

**Table 7:** Commercial devices which include Zynq devices.

Board Name	Manufacturer	Device	Price (USD)
Zybo Zynq-7000 ARM/FPGA SoC Trainer Board	Digilent	XC7Z010	189
Z-turn Kit	MYIR	XC7Z010	139
Z-turn Kit	MYIR	XC7Z020	159
PicoZed	Avnet	XC7Z010	149
PicoZed	Avnet	XC7Z015	259
PicoZed	Avnet	XC7Z020	199
PicoZed	Avnet	XC7Z030	369
MicroZed	Avnet	XC7Z010	169
MicroZed	Avnet	XC7Z020	245
ZedBoard	Avnet	XC7Z020	395
Arty Z7	Digilent	XC7Z020	209
RedPitaya	RedPitaya	XC7Z010	199

**Figure A1:**
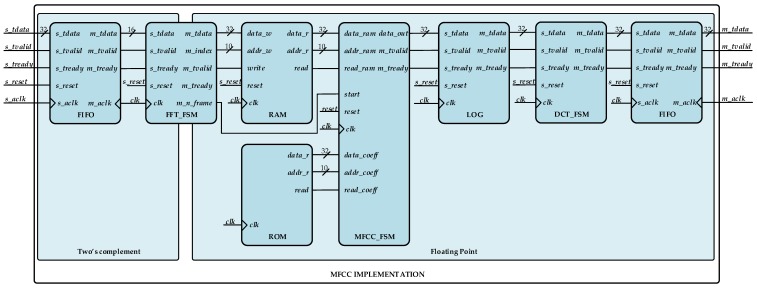
Main block diagram of the proposed Fiel Programmable Gate Array (FPGA) implementation.

**Figure A2:**
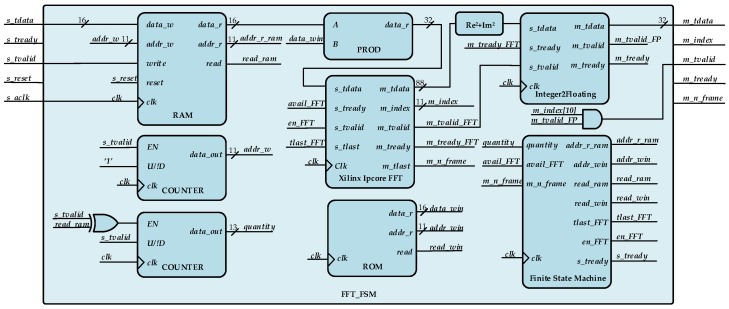
Algorithm description of the FFT_FSM subsystem, which includes the windowing and the power spectrum procedures.

**Figure A3:**
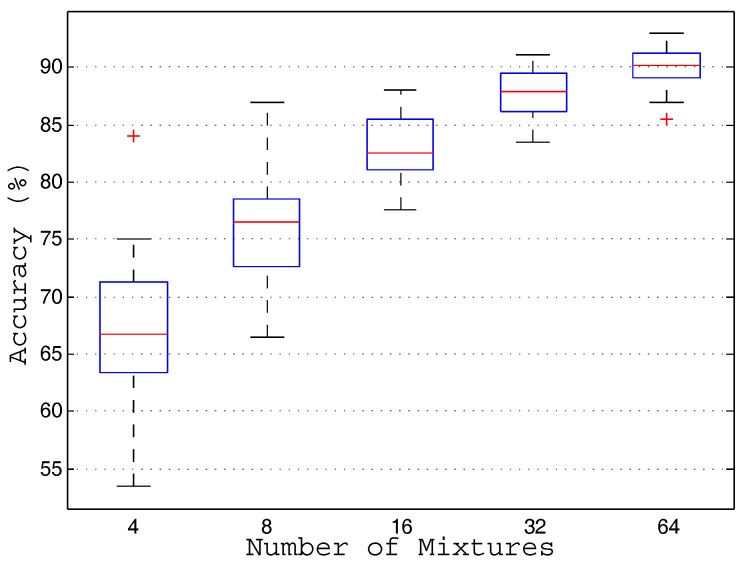
Boxplots showing the accuracy of the birdsong recognition algorithm considering a $2^{n}$ sweep of mixtures of the GMM.

## Abbreviations

The following abbreviations are used in this manuscript:

ADC	Analog to Digital Converter
AMBA	Advanced Microcontroller Bus Architecture
ARM	Advanced RISC Machines
AXI	Advanced eXtensible Interface
BRAM	Block RAM
CORDIC	COordinate Rotation DIgital Computer
CPU	Control Processing Unit
DAC	Digital to Analog Converter
DCT	Discrete Cosine Transform
DDR	Double Data Rate
DNN	Deep Neural Network
DSP	Digital Signal Processor
DTF	Discrete Fourier Transform
ESE	Efficient Speech Recognition Engine
FF	Flip-Flop
FFT	Fast Fourier Transform
FIFO	First Input First Output
FLOPS	Floating Point Operations Per Second
FPGA	Field Programmable Gate Array
FSM	Finite State Machine
GMM	Gaussian Mixture Model
GPU	Graphic Processor Unit
GTM	Grup de Recerca en Tecnologies Mèdia
IP	Intelectual Property
LPCC	Linear Predictive Cepstral Coefficients
LSTM	Long Short-Term Memory
LUT	Look-Up Table
MAC	Multiplier and Accumulator
MEC	Mobile Edge Computing
MFCC	Mel Frequency Cepstral Coefficients
MIDI	Musical Instrument Digital Interface
MLP	Multilayer Perceptron
PC	Personal Computer
PL	Programmable Logic
PS	Programmable System
PSD	Power Spectral Density
RAM	Random Access Memory
ROM	Read Only Memory
ROBIN	Recording and Observing Bird Identification Node
SIMD	Single Instruction-Multiple Data
SoC	System on Chip
STFT	Short Time Fourier Transform
SVM	Support Vector Machine
WASN	Wireless Acoustic Sensor Network

## Appendix A. MFCC Implementation on the FPGA in Detail

This Appendix provides information about the implementation of the MFCC computation in a Xilinx FPGA, following a top-bottom approach. In the first subsection, we start defining the whole algorithm. In the following subsections, we describe each block of the implementation. Please bear in mind that this is not a final implementation in a real hardware platform, but it provides us with approximate information about resource consumption of the algorithm. This data allows us to estimate the number of sensors that we would be able to process in parallel, besides giving us an idea of which devices could be used to do it.

### Appendix A.1. FPGA Implementation of the Algorithm

The entire implementation is depicted in Figure A1, where we can observe the modularity of the algorithm. It can be optimized on area or power consumption, and each of those parameters can be optimized individually in each of the components. As a description of the diagram, we would emphasise in (a) the pair of asynchronous FIFO memories in the input and output of the system working as buffers, separating clock domains and providing the AXI4 interface for the embedded system; (b) the FFT_FSM block, which reads a frame of 30 ms of data from the input FIFO (1440 samples), applies the Hamming windowing and obtains the power spectrum of this frame and stores it in a (c) RAM of 32 kb; (d) the MFCC_FSM reads data from the RAM and the (e) ROM memory of 1536 kb, where the filter bank coefficients are stored, to calculate the 48 values of the energy coefficients; and finally (f) the logarithmic conversion and the (g) DCT are applied storing 13 MFCC values in the output FIFO.

#### Appendix A.1.1. Description of the Power Spectrum Implementation

In the FFT_FSM block (see Figure A2), which calculates the Power Spectral Density (PSD) function of the input signal, the Fast Fourier Transform (FFT) has been implemented using the Xilinx [56] IP core Fast Fourier Transform v9.0 (San Jose, CA, USA). This IP core allows three arithmetic options for computing the FFT: (a) full-precision unscaled arithmetic; (b) scaled fixed-point and (c) block floating-point. Moreover, the user can choose between one of the following architecture options: (a) Pipelined Streaming I/O; (b) Radix-4 Burst I/O; (c) Radix-2 Burst I/O; and (d) Radix-2 Lite Burst I/O. The architecture options previously enumerated the execution time in number of clocks, and the required resources as estimated by Xilinx Vivado are depicted in Table A1. Please note that all those values are pre-implementation estimations and calculations done by the toolkit Vivado. The FFT IP core can be implemented using configurable logic blocks (CLB) instead of physical DSPs to obtain the desired trade-off between performance and required resources.

**Table A1:** Architecture options available using the Fast Fourier Transform v9.0 provided by Xilinx, its execution time and required resources for full-precision unscaled arithmetic.

Architecture Options	Execution Time	Estimated Required Resources		
	(# of Clocks)	DSPs	Block RAMs	LUTs
Pipelined Streaming Input/Output (I/O)	6285	50	25	–
Radix-4 Burst I/O	7297	36	18	–
Radix-2 Burst I/O	15,549	12	12	–
Radix-2 Lite Burst I/O	26,661	6	12	–

The input data provided by the analog to digital Ccnverters (ADCs) is usually supplied in a natural binary or two’s complement, so the FFT has been implemented in full-precision unscaled arithmetic. Moreover, the required resources for arithmetic operations is lower in two’s complement than in floating point. To do this, the Hamming windowing coefficients have been generated and stored in two’s complement, and the power computation of the spectrum has also been calculated in two’s complement. The overlap of 50% between frames is applied by the “Finite State Machine”, where the read pointer of the RAM memory is decreased 720 samples (15 ms) every frame from its current value. Finally, the data resulting of this block is converted into floating point arithmetic of single precision and stored in a RAM block. This conversion from two’s complement to floating point has been done to increase the dynamic range of the results without the need to increase the number of bits of the data buses in two’s complement. The implemented FFT uses Radix-2 Lite Burst I/O because the required resources are lower than the rest, while the execution time in this configuration is of 26,661 clock cycles, that with a clock frequency of 100 MHz would allow us to process an FFT frame of 30 ms in less than 300 μs. Moreover, as we show below, the bottleneck of the algorithm execution time is in the MFCC_FSM calculation.

#### Appendix A.1.2. Implementation of the MFCC Filter Bank Algorithm and the DCT

The implementation of the MFCC filter bank and the DCT algorithm follows a serial structure. The coefficients of both algorithms have been pre-calculated and stored in ROM memories, and the results are calculated by Multiplying and Accumulating (MAC) the values of the coefficients with the input data using a COordinate Rotation DIgital Computer (CORDIC) IP core from Xilinx able to do MAC operations in floating point arithmetic. Both memories have been implemented using Block RAM (BRAM), where the size of these memories can be calculated as $2^{m}\cdot n$, where *m* is the number of bits of the address bus and *n* the word size. Despite this implementation requiring a high amount of memory blocks in the filter bank of the MFCC because the number of coefficients is 1024 for each of the 48 filter banks, which is 1536 kb, the arithmetic resources such as DSP and LUT are lower than an implementation based on a real-time calculation of these coefficients.

## Appendix B. Evaluation of the Number of Mixtures of the GMM Algorithm

In this section, we present the results of the comparative study about the optimal number of mixtures of the GMM classifier in terms of accuracy and computational cost. This study has considered the following $2^{n}$ sweep: 4, 8, 16, 32 and 64 mixtures in order to find a good trade-off between accuracy and computational cost to implement the system in real-time. The reader is referred to Section 4.3 for the analysis of the computational cost, where we have argued that the computational cost varies linearly with the number of mixtures.

From Figure A3, we can observe that the recognition accuracy increases with the number of mixtures, showing a quasi-logarithmic pattern along the sweep. The highest averaged recognition values are obtained when considering 16, 32 and 64 mixtures (see Table A2). Specifically, an averaged accuracy of 87.8% is obtained with 32 mixtures, which increases 5% when compared to 16 mixtures, at the cost of doubling the computational cost. On the contrary, doubling again the computational cost with 64 mixtures, only yields 2.2% averaged accuracy increase (see Table A2). Therefore, considering these results, we select 32 mixtures as a good trade-off between recognition accuracy and computational costs.

**Table A2:** Averaged accuracy values of the GMM classifier considering a $2^{n}$ sweep from 4 to 64 mixtures.

**Number of Mixtures**	4	8	16	32	64
**Averaged Accuracy (%)**	67.1	75.7	82.8	87.8	90.0
